# Ontology-based construction of embroidery intangible cultural heritage knowledge graph: A case study of Qingyang sachets

**DOI:** 10.1371/journal.pone.0317447

**Published:** 2025-01-16

**Authors:** Yan Liang, Bingxue Xie, Wei Tan, Qiang Zhang

**Affiliations:** 1 Center for Studies of Ethnic Minorities in Northwest China, Lanzhou University, Lanzhou, China; 2 School of Literature, Huaiyin Normal University, Huaian, China; 3 Institute of Digital Humanities, Renmin University of China, Beijing, China; Kintampo Health Research Centre, GHANA

## Abstract

The fine-grained mining and construction of semantic associations within multimodal intangible cultural heritage (ICH) resources are crucial for deepening our understanding of their knowledge content and ensuring their systematic protection and transmission in the digital and intelligent era. This paper addresses the urgent need for the digital preservation and transmission of ICH resources. Following a review of current research on Qingyang sachets and ICH, the study introduces an ontology-based approach to constructing a semantic description model for the multimodal digital resources related to Qingyang sachets. By acquiring and processing multimodal resources concerning the craftsmanship and associated customs of Qingyang sachets, the study reorganizes the corresponding textual and visual knowledge. Utilizing knowledge graphs, the research explores multidimensional pathways for delivering knowledge services related to the multimodal digital resources of Qingyang sachets. Empirical research confirms the applicability and feasibility of the proposed semantic association scheme for multimodal ICH digital resources. The findings provide valuable insights for multidimensional organization and integration across scenarios, time periods, and resources within the ICH domain, offering a reference for digital solutions aimed at the systematic protection of ICH.

## Introduction

Intangible cultural heritage (ICH), also known as living culture, encompasses various non-material expressions such as skills, dances, and songs that have evolved over centuries through human activities. It serves as a testament to history, a product of human creativity, and a reflection of cultural diversity [1–6]. The importance of preserving ICH and recognizing its intrinsic value has gained widespread recognition in society. In the contemporary context, the pressing challenge is how to effectively transmit, protect, and utilize ICH resources, ensuring their creative transformation and innovative development. In 2003, UNESCO adopted the Convention for the Safeguarding of ICH, which significantly advanced the global efforts to protect and document ICH [1, 5–8]. Influenced by this convention, China’s Ministry of Culture and Tourism has established a national inventory of ICH since 2006, which now includes 1,557 ICH projects and 3,610 sub-projects [9]. The Qingyang sachet, the focus of this study, is among the significant items on this national ICH protection list. Qingyang sachets are traditional handcrafted embroidered items, known for their distinctive styles, shapes, patterns, and forms, and were once commonly used in daily life [10]. However, with the advent of technological advancements, much of this handicraft has been replaced, leading to the gradual decline of these traditional skills, which are no longer held in high regard. To prevent the permanent loss of these precious crafts, it is essential to employ modern digital technologies for the scientific and effective preservation of ICH, ensuring the sustainability of its protection and transmission in the digital era. On one hand, the presentation of Qingyang sachet ICH resources is transitioning from a single modality to a multimodal display that integrates text, images, and other forms. The discrete and unstructured multimodal resources create an urgent need for their effective organization and management. On the other hand, in the ongoing process of digitalization, there remains a pronounced tendency to prioritize technology over culture, resulting in insufficient attention to inheritors and artisans. Consequently, the cultural value and depth of knowledge embedded in Qingyang sachet ICH digital resources require further exploration and refinement. Therefore, at this stage, deepening the understanding of Qingyang sachet ICH resources and integrating them with digital technologies have become focal points in the phased development of digital resources within the Qingyang sachet ICH domain.

To promote the effective organization and utilization of multimodal digital resources related to Qingyang sachets, and to achieve fine-grained knowledge association within these resources, this study adopts a two-pronged approach. First, by integrating the theoretical framework of ICH protection systems, an ontology model is constructed to enable the detailed deconstruction of knowledge elements and the standardized definition of concepts and relationships within the multimodal digital resources of Qingyang sachets. This approach addresses the challenge of making these resources semantically describable. Second, the study combines formalized ontology representation with domain-specific knowledge graphs to achieve knowledge integration. The construction of a knowledge graph enables the visualization and relational representation of Qingyang sachets’ multimodal ICH digital resources, thereby revealing the complex and diverse relationships among knowledge elements and uncovering the rich cultural connotations embedded within these resources. From an application perspective, this approach facilitates knowledge discovery and application. This research offers a novel framework for the organization of Qingyang sachets’ multimodal ICH digital resources, providing new pathways for the promotion, transmission, and utilization of traditional embroidery culture. Additionally, it serves as a valuable reference for the digital preservation and development of ICH resources.

Currently, the organization of information related to Qingyang sachets primarily relies on books or unstructured, dispersed data available online, lacking a standardized or structured corpus. Multimodal knowledge graphs, a prevalent technique in the digital humanities for organizing and storing knowledge, offer an effective means to systematize fragmented information and present it clearly through visualizations. This approach would facilitate more efficient knowledge management and application. This paper, grounded in the digital humanities perspective, addresses the challenges posed by the diverse and complex data sources involved in organizing ICH resources. By employing multimodal knowledge graphs—a representative technology in the digital humanities—and focusing on Qingyang sachets as the research subject, this study adopts a top-down approach to construct a multimodal knowledge graph for Qingyang sachets. The research delves into the digital organization, storage, and application of Qingyang sachets, with the ultimate aim of facilitating knowledge discovery related to this traditional craft.

## Related work

In this section, we will review the related work on Qingyang sachet research as well as the progress in knowledge graph construction within the cultural heritage field, extending beyond the scope of ICH.

### Research on Qingyang sachets

Qingyang sachets are traditional folk embroidery items crafted from fabric and silk threads, filled with herbs that emit a fragrant scent. These sachets are believed to serve both practical functions, such as repelling mosquitoes, and symbolic purposes, such as bringing blessings. Originally created by artisans to meet the spiritual and practical needs of daily life [11], Qingyang sachets have deep historical roots. According to historical records, approximately 2,600 years ago, Qibo, a legendary figure regarded as the founder of traditional Chinese medicine, taught people to wrap medicinal herbs in fabric pouches to prevent snake and insect bites and to ward off epidemics. This practice is considered the earliest prototype of the Qingyang sachet, which has been passed down through generations to the present day [12]. However, current research on Qingyang sachets remains somewhat limited, primarily focusing on three aspects:

1. Research on Qingyang sachet artisans: Miao [13] conducted in-depth interviews with 10 folk artisans, providing insights into their life experiences and creative backgrounds. Wang [14] offered detailed introductions to well-known folk artisans in Qingyang.
2. Research on Qingyang sachet works: Cao [15] categorized hundreds of sachet works into three major groups: wearable accessories, legendary stories, and daily necessities. Each category was analyzed to interpret the cultural significance embedded in these embroidered items. Wang and Zhang [16] have collected numerous exquisite images of sachets, including items such as pincushions, pillow tops, dudou (traditional undergarments), and embroidered shoes, to provide a comprehensive overview of the craftsmanship involved in sachet and embroidery production. They summarize the production process into six main steps: designing patterns, cutting fabric, embroidering, sewing sachets, filling with herbs, and sealing and decorating.
3. Research on the artistic style and cultural connotations of sachets: Zhao [17], after understanding the history of sachets, conducted a study on their artistic design and explored the economic value. He believed that sachets not only create numerous employment opportunities but also promote the development of tourism. Liang [18] provided an interpretation of the symbolic metaphors associated with sachets, suggesting that elucidating the cultural symbolism of sachets contributes to a deeper understanding of their cultural value and strengthens efforts toward their preservation and transmission. For instance, frogs are regarded as sacred beings by the local people, and because of their strong reproductive capacity, they are also considered symbols of fertility and proliferation. It is believed that worshipping frogs can bring good fortune and a large, thriving family. Similarly, fish are seen as symbols of fertility. The dragon and phoenix motifs, symbols of authority and auspiciousness in Chinese culture, represent marital happiness and familial harmony when appearing together on a sachet.

The existing research on Qingyang sachets predominantly adopts a humanistic perspective, with a noticeable lack of studies focused on their digitization and knowledge integration. Therefore, constructing a knowledge graph for sachets to systematically represent and organize related knowledge is of significant importance for the digital preservation, dissemination, and innovative development of sachet culture. Based on the sachet knowledge graph, it would be possible to develop intelligent systems for showcasing sachet craftsmanship, smart tools to assist in designing sachet patterns, and public cultural outreach platforms. These applications would effectively promote the dissemination and practical application of sachet artistry.

### Research on knowledge graphs for ICH

With the advent of the digital and intelligent era, semantic technologies such as ontology, linked data, and knowledge graphs have been increasingly applied to the knowledge organization of cultural heritage digital projects [7]. These technologies have been widely utilized in internationally renowned digital humanities projects, such as the Europeana project [19] in the European Union and the CultureSampo project [20] in Finland. On a practical research level, international studies have focused on applied use cases. For example, Kim et al. [21] proposed an innovative augmented reality application based on ontology to provide contextual information for cultural heritage sites. Isa et al. [22] employed ontology for domain knowledge modeling of the Terengganu brassware craft. Lombardo [23] designed an ontology model for theatrical culture, creating a visual graph that incorporates character emotions and intentions. Thanh [24] conducted knowledge modeling of traditional Vietnamese dance using ontology. On the basis of sorting out the data status of galleries, libraries, archives and museums across Europe, Charles et al. [25] designed a user-oriented digital library that provides multilingual retrieval by using knowledge graph technology. It provides the possibility for digital organization of cultural heritage. Additionally, Coladangelo [26] developed a dance terminology lexicon and designed a domain ontology to preserve and disseminate cultural heritage information related to North American folk dance.

In China, significant progress has been made in the knowledge organization of ICH resources, both in theory and practice. From a macro perspective, Zhang et al. [27] have conducted research on knowledge organization models and the construction of humanistic graphs for ICH texts. Fan et al. [28] have focused on building knowledge graphs for ICH. On a practical level, several case studies have been conducted. For example, He et al. [29] constructed an ontology-based knowledge base using Hezhe ethnic group ICH resources as a case study, while Wang et al. [30] developed a knowledge graph for Yuanqu (Yuan Dynasty poetry) based on an ontology model. Driven by advancements in digital technologies, digital ICH resources have garnered more attention compared to traditional resources. Some studies have focused on ICH inheritors, such as Li’s [31] research on organizing digital resources for the personal archives of ICH inheritors using metadata and ontology. Others have explored multimodal ICH digital resources, such as Zhu et al. [32], who conducted research on linked data storage and publishing based on the construction of an ontology for the semantic information of ICH images.

In summary, the current trend in ICH knowledge organization is shifting from external, coarse-grained approaches to the integration of internal characteristics of information resources. The focus of knowledge organization has evolved from single-form resources to diversified multimodal digital resources. Semantic web technologies such as ontology, linked data, and knowledge graphs have demonstrated superior capabilities in the aggregation, association, and discovery of ICH knowledge. These technologies provide valuable insights for advancing the protection of Qingyang sachets from mere “digitization” to “datafication” and “intelligent” approaches. Additionally, they offer new strategies for empowering smart data and facilitating the generation of cultural memory.

## Overview of the framework

**Fig 1:**
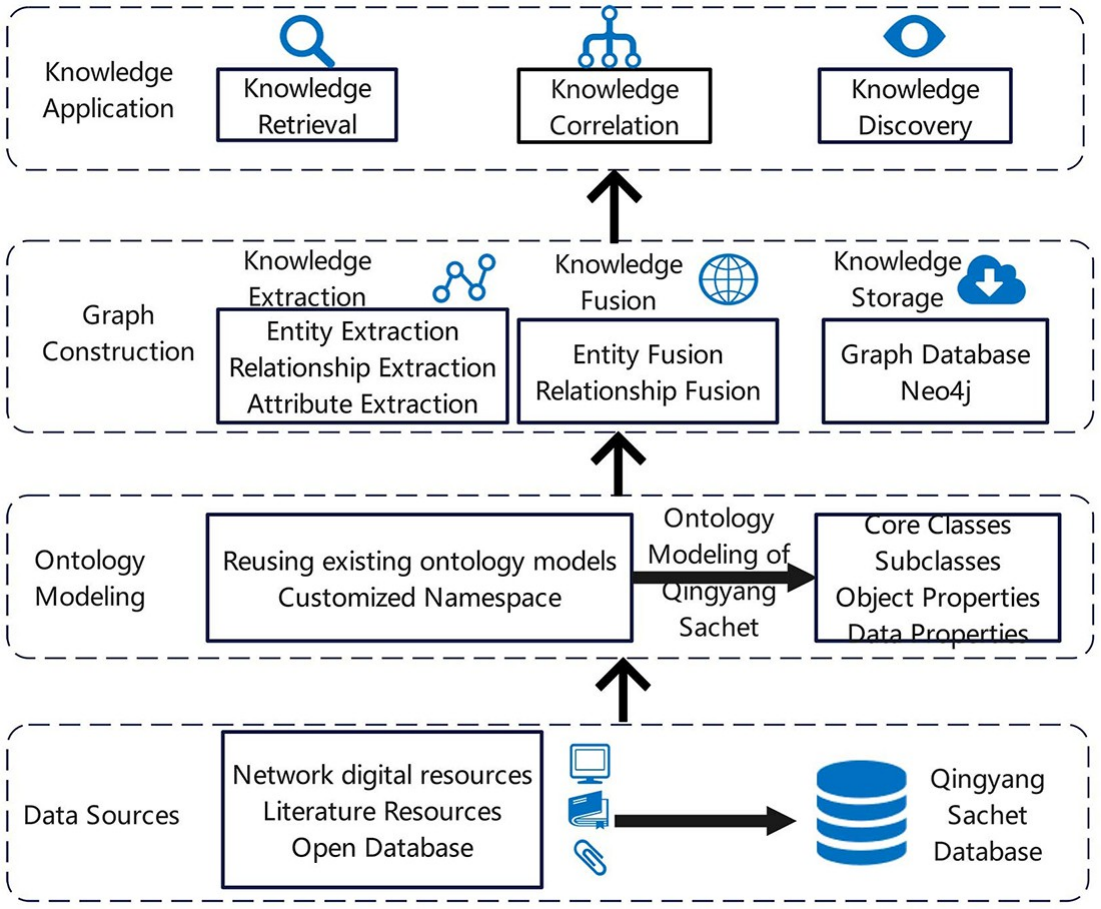
Research framework.

The primary focus of this study is the systematic and structured organization of Qingyang sachets, with the aim of constructing a domain ontology for Qingyang sachets. This ontology will facilitate the fine-grained description of basic information and knowledge associations related to the sachets. By building a multimodal knowledge graph, the study seeks to achieve visual representation and knowledge discovery, thereby supporting the preservation, transmission, and promotion of Qingyang sachets. Therefore, the study proposes the design of an ontology model for Qingyang sachets and, based on this model, conducts an empirical study using the multimodal knowledge graph. The objective is to enable visual display, knowledge retrieval, and other applications of the knowledge content. The specific research approach, as illustrated in Fig 1, consists of four main components: data sources. ontology modeling, graph construction, and knowledge application.

First, data sources are the foundation for the entire graph construction process. The collected multi-source heterogeneous data must be structured, including structured data from open-source databases, semi-structured data from online information resources, and unstructured data from books and literature.

Next, the study proposes reusing existing ontologies to analyze the composition and characteristics of the collected Qingyang sachet knowledge. This involves defining the concepts, relationships, and properties of Qingyang sachets. Ontology theory is then applied to abstract and generalize the Qingyang sachet data at a logical level, thereby achieving ontology modeling and constructing the schema layer of the knowledge graph.

A knowledge graph is built using triples such as “entity-relationship-entity” and “entity-property-property value,” representing entities in the physical world and the relationships between them, forming a structured semantic knowledge base [33, 43]. Depending on the specific needs and application scenarios, the construction of a knowledge graph necessarily relies on various technologies. This study primarily focuses on knowledge graph technologies such as knowledge extraction, knowledge fusion, and knowledge storage. The collected Qingyang sachet instances are imported into the Neo4j graph database, enriching the data layer of the Qingyang sachet knowledge graph. This allows for the visual representation of the entire set of instances and facilitates the exploration of potential knowledge discoveries.

Finally, the ultimate goal of constructing the multimodal knowledge graph is to enhance knowledge application. From the perspective of knowledge association, this study utilizes the graph for knowledge presentation and retrieval, enabling users to gain a multidimensional and fine-grained understanding of Qingyang sachets and uncover the hidden knowledge behind them.

## Case study

### Data sources and processing of the Qingyang sachets

The multimodal digital resources include not only textual information but also images, videos, and audio, encompassing a wide variety of media forms. Qinyang sachet holds a significant position in the cultural heritage of Chinese handcrafted embroidery. As such, various elements related to embroidery craftsmanship have been included. The multimodal ICH digital resources of Qingyang sachets constitute an integrated multimedia collection of text, images, videos, and audio, aimed at promoting the preservation, transmission, and dissemination of this ICH.

The specific knowledge elements of Qingyang sachets’ multimodal ICH digital resources used in this study are primarily derived from several authoritative sources. These include *A Hundred Freehand Interpretations of Qingyang Sachets* edited by Cao [15], *Qingyang Sachets* by Yu [10], *The Illustrated Dictionary of Qingyang Sachet Folk Cultural Products* [34], *The Embroidery Art of Qingyang Sachets* by Dong [35], *Qingyang Sachets Embroidery* [11] compiled by the Qingyang Cultural Center, and the catalog of sachet collections from the Qingyang Museum [36], including online resources from the Qingyang Museum website. These sources ensure the accuracy and credibility of the data collected.

**Fig 2:**
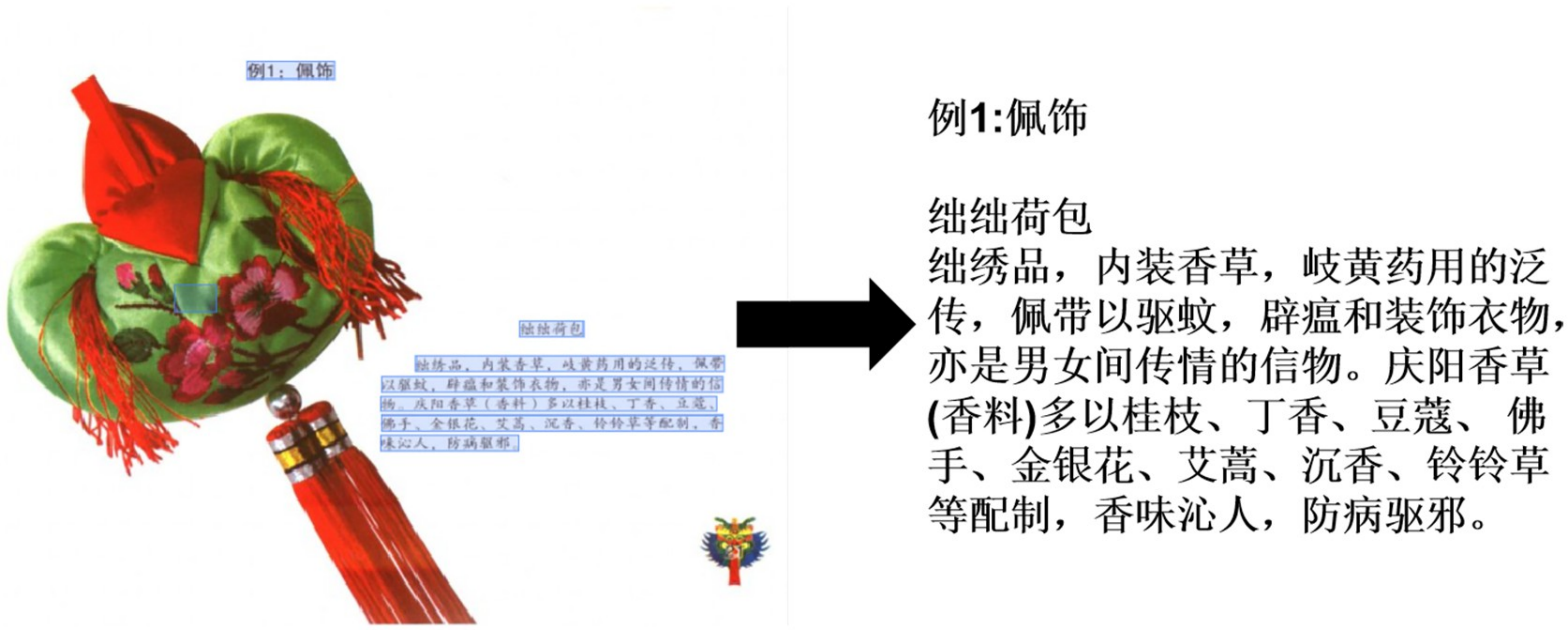
PP-Chatv3 OCR recognition example.

For physical paper documents, OCR technology is first used to digitize images into text data. Specifically, this study utilizes PP-Chat OCRv3 to extract text from images. PP-Chat OCRv3 is an intelligent document and image analysis solution developed by Baidu’s PaddlePaddle, combining LLM with OCR technology. It is equipped with language-specific training data for Chinese text to enhance recognition accuracy. The recognition results of PP-Chat OCRv3 are shown in Fig 2. After recognition, the text data is stored in a database. Data preprocessing is then performed to reduce noise and interference, enhance image contrast, and improve data quality. The data cleaning steps include removing duplicates, standardizing format inconsistencies (e.g., date formats), and filtering out irrelevant records based on predefined criteria. For errors encountered during preprocessing, the built-in text correction function of the OCR software is used, along with manual correction, to ensure the accuracy of the foundational data. Finally, relevant data concerning the patterns, types, and content of Qingyang sachets are manually extracted and saved in a tabular format. As for resources from encyclopedias and official websites, they are obtained directly through web scraping, cleaned, and then stored.

In addition to text data, we conducted fieldwork in Qingyang from May 2023 to July 2024, visiting various areas across Qingyang, Gansu Province. Using professional equipment, we collected over 1500 original images of sachet artifacts (supplementary material 1). During the image preprocessing phase, we first manually filtered the original images based on the completeness and clarity of the patterns, removing low-quality images. Then, we used Photoshop to extract individual pattern samples from the original images, cropping along the pattern boundaries. After preprocessing, the final data collection is summarized in Table 1.

**Table 1:** Data collection volume from different sources.

Data sources	Data quantity	
	Images(sheet)	Text(item)
Fieldwork data resources	1528	624359
Encyclopaedias	456	334
OCR-recognized text	0	574

Source: Authors’ own work.

It should be noted that this research has received approval from the Ethics Review Committee of Lanzhou University and is in accordance with the UNESCO Declaration on the Protection of Cultural Heritage [5]. All participants took part voluntarily, with full informed consent. To protect participants’ privacy, strict anonymization measures were implemented during data collection and processing. All personal information was anonymized, and encryption techniques were used to store the data, ensuring protection against unauthorized access. In the collection of ICH data, we fully respected local cultural customs and traditions. Local community members were invited to participate in the data collection process, and their feedback and suggestions were taken into consideration.

### Ontology modeling

Ontology construction, as the schema layer of a knowledge graph, plays a crucial role in constraining conceptual relationships and defining concept attributes [37, 48]. As Gruber stated, “An ontology is an explicit specification of a conceptualization. [38]” Ontology provides constraints and standardization for the data layer and serves as one of the main approaches to knowledge representation and organization, with applications across a wide range of fields. With the advancement of ontology construction research, various methodologies have emerged, such as the TOVE method, METHONTOLOGY, the skeletal method, KACTUS engineering, the seven-step method, IDEF5, and approaches based on thesauri [39]. Given that this study aims to construct a domain ontology for Qingyang sachets, the seven-step method, known for its advantages in domain applicability, has been chosen as the primary reference methodology for this research.

#### Conceptual framework construction

According to the requirements of the seven-step method for ontology construction, the first step is to clearly define the scope of conceptual descriptions for the domain of Qingyang sachets. Based on the previous analysis of Qingyang sachet knowledge, the primary ontology models relevant to this study include the Friend of a Friend (FOAF) [40] ontology and the CIDOC Conceptual Reference Model (CIDOC-CRM) [41] developed by the International Committee for Documentation of the International Council of Museums. FOAF is mainly used for describing interpersonal relationships and personal information through an RDF vocabulary, whereas CIDOC-CRM, as an international standard for describing cultural heritage information, offers extensive domain coverage and openness, making it suitable for the description of ICH resources. The mature architecture of CIDOC-CRM facilitates semantic mapping and alignment with other ontologies, promoting cross-disciplinary and cross-linguistic knowledge sharing. Additionally, CIDOC-CRM’s high level of specialization in the cultural heritage domain gives it advantages over other ontology models.

However, existing ontology models cannot fully meet the needs for fine-grained descriptions of Qingyang sachet knowledge in this research. Therefore, building on existing ontologies, this study reuses classes related to time, place, person, and event while developing a custom Qingyang Sachet Ontology (QSO). QSO includes six custom classes: Qingyang Sachet (QSO:QingyangSachet), Craftsmanship (QSO:Craftsmanship), Material (QSO:Material), Pattern (QSO:Pattern), Function (QSO:Function), and Cultural Significance (QSO:CulturalSignificance).

Qingyang sachets are an important part of China’s national-level ICH in handcrafts. Building on the naming and classification standards established for existing hand-embroidery-related intangible cultural heritage ontologies [7, 27–29, 32, 33, 42], we identified that key elements of handcraft embroidery projects include works, craftsmanship, materials, patterns, functions, and cultural significance. Furthermore, the accuracy, completeness, usability, and consistency of information are fundamental standards for ontology construction [37, 45]. Therefore, we have defined six custom classes, resulting in a total of ten categories to fulfill the abstraction requirements for conceptualizing Qingyang sachet knowledge. The inter-class hierarchical relationships are shown in Table 2. In the following sections, regarding the construction and application of the Qingyang sachet knowledge graph, we conducted simulated tests on the ontology model, such as testing the knowledge discovery and knowledge retrieval functions. The results show that the constructed Qingyang sachet ontology model is both valid and reliable. Due to space limitations, we plan to conduct research on practical case studies in the future to incorporate feedback from usage scenarios.

**Table 2:** Inter-class hierarchical relationships.

Core classes	Subclasses
QingyangSachet(QSO:QingyangSachet)	None
Time(Crm:timespan)	Time Specific(Crm:timeSpecific) Time Abstract(Crm:timeAbstract)
Place(Crm:place)	None
Person(Foaf:person)	Folk Craftsmen(QSO:folk craftsmen) Inheritors(QSO:inheritors) Researchers(QSO:researchers) Promoters(QSO:promoters) Apprentices(QSO:apprentices)
Event(Crm:event)	None
Craftsmanship(QSO:craftsmanship)	Designing Patterns(QSO:designing patterns) Cutting Fabric(QSO:cutting fabric) Embroidering(QSO:embroidering) Sewing Sachet(QSO:sewing sachet) Filling with Herbs(QSO:filling with herbs) Sealing and Decorating(QSO:sealing and decorating)
Material(QSO:material)	Fabric(QSO:fabric) Fillings(QSO:fillings) Threads(QSO:threads) Herbs(QSO:herbs) Embroidery Needles(QSO:embroidery needles)
Pattern(QSO:pattern)	Animal Patterns(QSO:animal patterns) Plant Motifs(QSO:plant motifs) Figures(QSO:figures) Symbols(QSO:symbols)
Function(QSO:function)	Decoration(QSO:decoration) Exorcism(QSO:exorcism) Health Care(QSO:health care) Blessings(QSO:blessings)
Cultural Significance (QSO:culturalsignificance)	Nature Worship(QSO:nature worship) Fertility Worship(QSO:fertility worship) Totem Worship(QSO:totem worship) Prosperity and Auspiciousness (QSO:prosperity and auspiciousness) Peace and Well-being (QSO:peace and well-being)

Source: Authors’ own work.

(1) Qingyang Sachet Class

The Qingyang Sachet class (QSO:QingyangSachet) refers to the works of Qingyang sachets, which are the core subject of Qingyang sachet resources. This class serves as the central element in the ontology construction, linking with other classes to collectively form a semantic knowledge network within the domain of Qingyang sachet knowledge.

(2) Time Class

The Time class (crm:TimeSpan) primarily encompasses all time-related information associated with Qingyang sachets, as well as people and events related to them. Based on how time is structured, the Time class is further divided into two subclasses: AbstractTime and SpecificTime. Abstract Time represents indeterminate time periods, such as the late Qing Dynasty, while Specific Time refers to precise time points, such as the year 1930.

(3) Place Class

The Place class (crm:place) covers spatial information related to the Qingyang sachet, person, and event. For Qingyang sachets, this primarily refers to the locations where they are created and exhibited. For a person, it includes places of birth and death, while for an event, it focuses on the places where the event occurred. The specific data properties of the Place class include details such as place names and geographic coordinates.

(4) Person Class

The Person class (Foaf:person) comprises five subclasses: Folk craftsmen (QSO:folk craftsmen), Inheritors (QSO:inheritors), Researchers (QSO:researchers), Promoters (QSO:promoters), and Apprentices (QSO:apprentices). Folk craftsmen refer to artisans who specialize in designing and creating sachets. Inheritors are individuals officially recognized as representative inheritors of ICH, possessing the knowledge, skills, and techniques to preserve and pass down the craft. Researchers are scholars who study the history, culture, and craftsmanship of sachets. Promoters are individuals who disseminate knowledge of sachets and promote their culture and craftsmanship through community activities, online tutorials, and other methods. Apprentices are individuals who acquire sachet-making skills through observation, imitation, and practice. The data properties for this class include the person’s name, achievements (honors), and level.

(5) Event Class

The Event class (crm:event) represents a collection of relevant events associated with Qingyang sachet works. This class has object property relationships with the Qingyang Sachet, Time, and Place classes.

(6) Craftsmanship Class

The Craftsmanship class (QSO:Craftsmanship) represents the craftsmanship of Qingyang sachets. Although the craftsmanship of each sachet may vary, the overall process can generally be divided into six steps: designing patterns, cutting fabric, embroidering, sewing sachets, filling with herbs, and sealing and decorating. There is an object property relationship between the Craftsmanship class and the Qingyang Sachet class.

(7) Material Class

The Material class (QSO:material) refers to the various materials used in the making of Qingyang sachets, such as fabric, fillings, threads, herbs, and embroidery needles. This class is related to the Qingyang Sachet class through object properties.

(8) Pattern Class

The Pattern class (QSO:pattern) represents the designs embroidered on the sachets. These patterns can be categorized into animals, plants, figures, and symbols. This class is related to the Qingyang Sachet class through object properties.

(9) Function Class

The Function class (QSO:function) is a crucial aspect of Qingyang sachets, encompassing a range of practical and cultural functions. These functions are mainly reflected in four areas: decoration, exorcism, health care, and blessings. This class is related to the Qingyang Sachet class through object properties.

(10) Cultural Significance Class

The Cultural Significance class (QSO:culturalsignificance) highlights that Qingyang sachets are not merely handicrafts but also cultural symbols that carry deep cultural connotations and embody folk traditions. The cultural significance is mainly reflected in five aspects: nature worship, fertility worship, totem worship, prosperity and auspiciousness, and peace and well-being. It is related to the Qingyang Sachet class through object properties.

#### Object property

After systematically analyzing the knowledge related to Qingyang sachets and defining the core classes of the Qingyang Sachet Ontology Model, it is necessary to further define the properties of these classes to clarify the hierarchical structure of the ontology. In the ontology, class properties are categorized into two types: object properties and data properties. Object properties are primarily used to describe relationships between classes, with both the domain and range being classes. Establishing object properties facilitates semantic associations and knowledge discovery within the sachet knowledge graph. On the other hand, data properties describe the inherent attributes of a class, where the domain is the class itself, and the range is a specific data type [42]. The ontology model constructed in this study includes a total of 10 core class concepts and 15 object properties, as shown in Table 3.

**Table 3:** Object properties relationship table.

Meaning	Object properties	Domains	Ranges
Qingyang Sachet and Time	QSO:createTimeOf QSO:exhibiteTimeOf	QSO:QingyangSachet	Crm:timespan Crm:timespan
Qingyang Sachet and Place	QSO:wasCreatedIn QSO:isExhibitedIn	QSO:QingyangSachet	Crm:place Crm:place
Qingyang Sachet and Figures	QSO:createdByCraftsmen QSO:inheritedFrom QSO:isPromotedBy QSO:isStudiedBy QSO:learnFrom	QSO:QingyangSachet	Foaf:person
Qingyang Sachet and Events	QSO:relatedTo	QSO:QingyangSachet	Crm:event
Qingyang Sachet and Craftsmanship	QSO:craftsmanshipUses	QSO:QingyangSachet	QSO:craftsmanship
Qingyang Sachet and Materials	QSO:consistsOfMaterial	QSO:QingyangSachet	QSO:material
Qingyang Sachet and Patterns	QSO:embroideredWithPattern	QSO:QingyangSachet	QSO:pattern
Qingyang Sachet and Functions	QSO:fulfillsFunction	QSO:QingyangSachet	QSO:function
Qingyang Sachet and Cultural Significance	QSO:carriesCulturalsignificance	QSO:QingyangSachet	QSO:culturalsignificance
Figures and Time	QSO:birthDate QSO:deathDate	Foaf:person	Crm:timespan Crm:timespan
Figures and Place	QSO:birthPlace QSO:deathPlace	Foaf:person	Crm:place
Figures and Events	QSO:haveEventOf	Foaf:person	Crm:event
Figures and Figures	QSO:mentorship QSO:colleagueRelation	Foaf:person	Foaf:person
Events and Time	QSO:beginTime QSO:endTime QSO:occurTime	Crm:event	Crm:timespan
Events and Place	QSO:occurPlace QSO:endPlace	Crm:event	Crm:place

Source: Authors’ own work.

#### Data property

The relationships between classes are explained through object properties, while the descriptive information within a class is expressed through data properties [43]. Defining data properties for the Qingyang Sachet Ontology enriches the description of Qingyang sachet knowledge instances, thereby extending the meaning of these instances. The data properties used in this study are detailed in Table 4.

**Table 4:** Data properties table.

Domains	Attribute meaning	Attribute name	Ranges
QSO:QingyangSachet	Name Image Video Audio Size Color Source Uses History Heritage situation Heritage value	QSO:name QSO:image QSO:video QSO:audio QSO:size QSO:color QSO:source QSO:uses QSO:hasHistory QSO:heritageSituation QSO:heritageValue	xsd:string xsd:anyURI xsd:anyURI xsd:anyURI xsd:string xsd:string xsd:string xsd:string xsd:string xsd:string xsd:string
Crm:timespan	Dynasty Festival Year Month Day Age Experience	QSO:dynasty QSO:festival QSO:year QSO:month QSO:day QSO:age QSO:yearsOfExperience	xsd:dateTime
Crm:place	Latitude and Longitude Place Name	QSO:latitude and longitude QSO:placename	xsd:string
Foaf:person	Name Achievements/Honors Level	QSO:name QSO:achievements/honors QSO:level	xsd:string
Crm:event	Event Name Event Content	QSO:name QSO:content	xsd:string
QSO:craftsmanship	Craftsmanship Name	QSO:craftsmanshipName	xsd:string
QSO:material	Material Name	QSO:materialName	xsd:string
QSO:pattern	Pattern Name Style Popularity	QSO:patternName QSO:style QSO:popularity	xsd:string
QSO:function	Function Name	QSO:content	xsd:string
QSO:culturalSignificance	Cultural significance content Cultural significance explanation	QSO:content QSO:explanation	xsd:string

Source: Authors’ own work.

**Fig 3:**
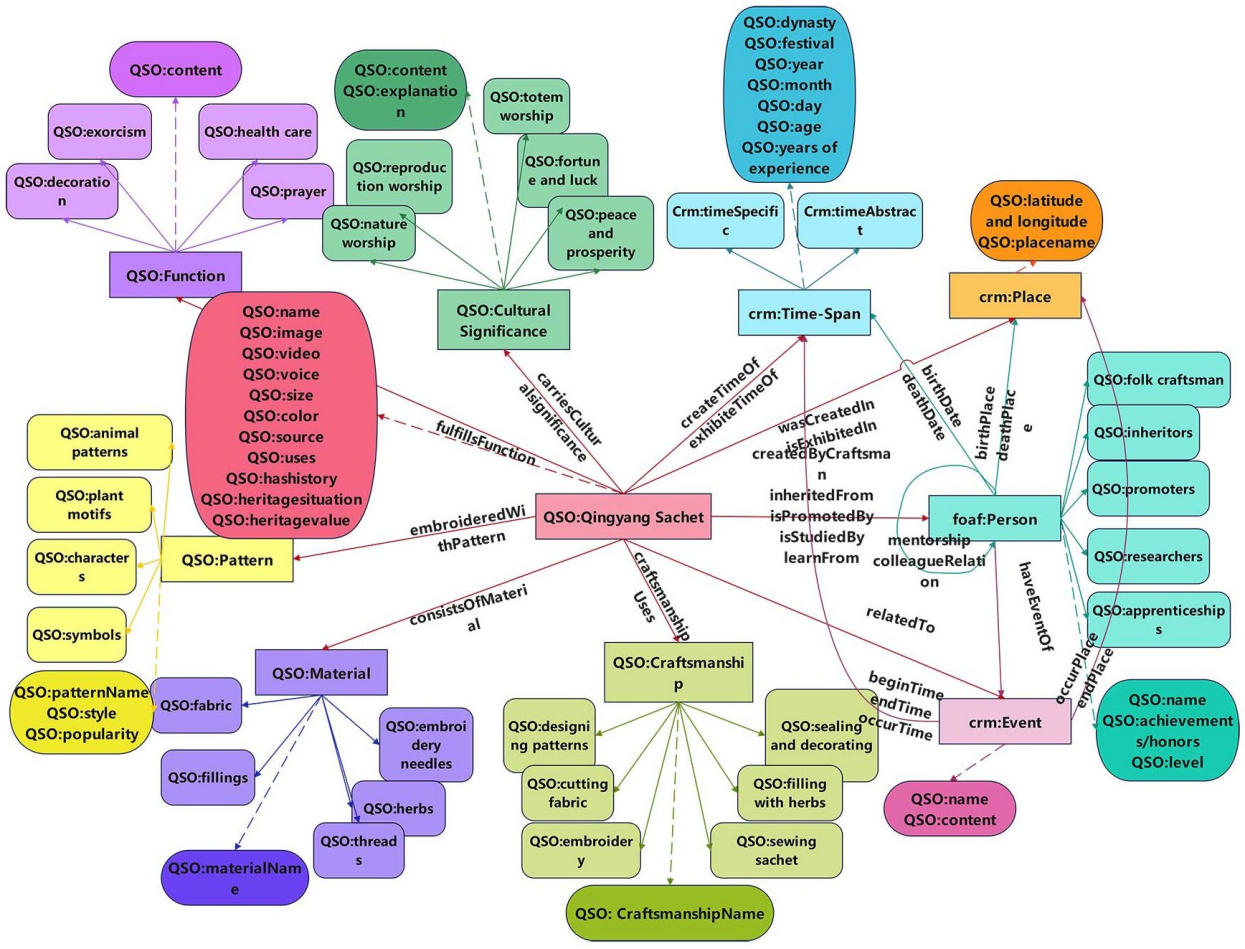
Qingyang sachet knowledge ontology model.

After designing the vocabulary for entities, relationships, and properties, engineering modeling was carried out using Protégé software. By defining rules and importing instance data, RDF data was generated, resulting in the fine-grained organization of Qingyang sachets’ multimodal ICH digital resources. Given that ontology construction may be subject to subjective limitations, leading to potential errors and redundancies, evaluation and optimization of the ontology are required. This can be achieved from two perspectives: referencing existing ontology models for ICH related to hand-embroidery and conducting case testing. Ultimately, the knowledge ontology model for Qingyang sachets developed in this study, as shown in Fig 3, includes 10 core concept classes, 7 subclasses, 15 object properties, and 33 data properties. The construction of this ontology model enables an in-depth analysis and comprehensive description of the concepts related to Qingyang sachets’ multimodal ICH digital resources, laying a foundational framework for the instantiation of the multimodal knowledge graph of Qingyang sachets in subsequent sections.

As illustrated in Fig 3, the ontology relationship mapping diagram displays the hierarchical relationships between the various properties of Qingyang sachets. This diagram uses different nodes and connections to clearly present the relationships and hierarchical structure among the properties. Specifically, right-angled rectangular icons represent the 10 core concept classes, while rounded rectangular icons correspond to the 33 data properties associated with these classes. Solid lines indicate object property relationships between classes, whereas dashed lines represent the data property relationships within the core concept classes.

### Construction of the Qingyang sachet knowledge graph

By leveraging the semantic associations established between various concepts at the schema level, this study conducts an empirical analysis of the knowledge graph using Qingyang sachet knowledge as an example. The study involves knowledge extraction, fusion, and storage, with the aim of disseminating domain knowledge, facilitating user queries, and enhancing the alignment between sachet resources and user search needs through semantic associations. This approach ultimately achieves comprehensive, network-based recommendations for Qingyang sachet knowledge and enables multidimensional knowledge discovery.

#### Knowledge extraction

**Fig 4:**
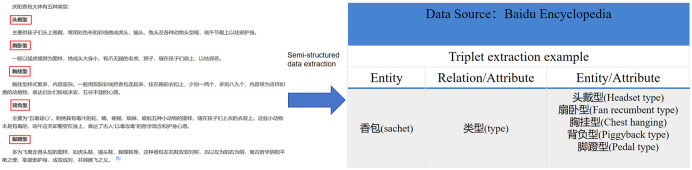
Example of knowledge extraction.

Knowledge extraction is the first step in constructing a knowledge graph. It primarily involves extracting structured data, such as entities, relationships, and properties, from diverse and heterogeneous data sources and transforming them into entity-relationship triples [44]. In this study, we first organize and extract entity properties, relationship properties, and data properties specific to Qingyang sachets. Specifically, entity extraction involves the accurate description of concepts, such as the identification and understanding of information related to Qingyang sachets, time, place, people, events and other relevant entities. Relationship properties extraction interprets the connections between entities, identifying the semantic relationships between entities, particularly through the extraction of triples, such as “Qingyang sachet—event—person” or “Qingyang sachet—event—place.” Data attributes pertain to the extraction of relevant properties of entities, enhancing the precision and comprehensiveness of entity descriptions. For instance, the data properties for a person might include name, achievements (honors), and level. In our preliminary work, the research team has already collected relevant textual data comprising 624,359 characters and 1,528 image resources. From these, 2,285 triples have been manually extracted. Additionally, online encyclopedic resources are used as supplementary data sources, and the corresponding knowledge extraction process is illustrated in Fig 4.

#### Knowledge fusion

Knowledge fusion is the process of integrating heterogeneous data from different sources within a unified framework, representing a high-level knowledge organization process. Generally, knowledge fusion involves the integration of concepts, relationships, and properties at the ontology layer, as well as the fusion of instances and properties values at the data layer [45]. Through knowledge fusion, ontologies can be shared, and their concepts and properties extended, thereby maximizing their utility. After knowledge extraction, the data may contain overlapping or erroneous information that requires reorganization to eliminate inconsistencies and ambiguities. For example, an entity may have multiple expressions, or a specific term might refer to multiple different entities. To resolve the diversity and ambiguity of entities, entity disambiguation can be applied to address cases where different names refer to the same entity.

**Fig 5:**
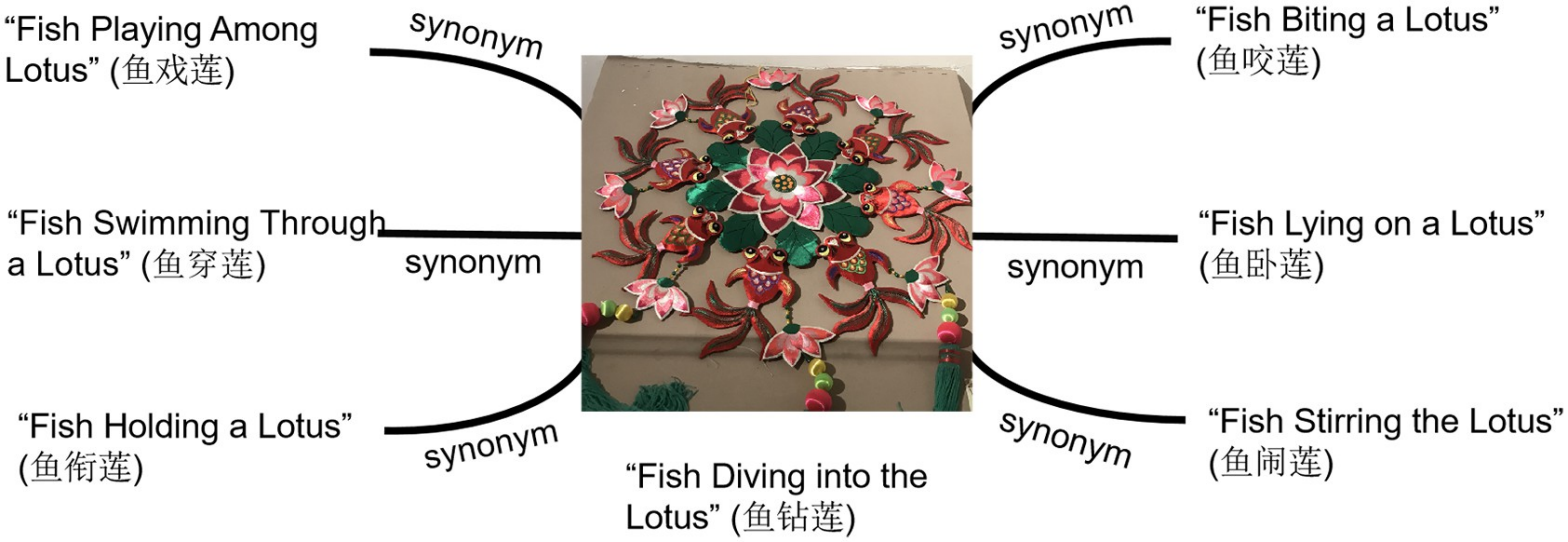
Entity alignment diagram.

For example, the Qingyang sachet work *Fish Diving into the Lotus* (鱼钻莲) is often recorded in textual data as *Fish Diving into the Lotus*, but it is commonly referred to as *Fish Playing Among Lotus* (鱼戏莲), *Fish Swimming Through a Lotus* (鱼穿莲), *Fish Lying on a Lotus* (鱼卧莲), *Fish Stirring the Lotus* (鱼闹莲), *Fish Holding a Lotus* (鱼衔莲), *or Fish Biting a Lotus* (鱼咬莲) in the oral accounts of folk craftsmen or inheritors. Although the names differ, they refer to the same work. Such entity types cannot be fused using conventional algorithms, as the similarity determined by the algorithm does not align with the actual situation. Therefore, the ambiguity associated with entities is primarily resolved by manually constructing custom synonym dictionaries. By collecting and analyzing a large corpus of data, a domain-specific synonym dictionary was developed, containing various names and references for the same entity. This allows for the unification of entities with different names during the knowledge fusion process, thereby addressing the issue of entity ambiguity. Fig 5 illustrates the alignment of the entity *Fish Diving into the Lotus* sachet. For relationship names, the cosine similarity measurement method was adopted. This method assesses the similarity between different expressions by calculating the cosine value between text vectors, effectively identifying and integrating different expressions that describe the same relationship. In practice, the relationship names are first converted into vector representations, and then the extended version of Harbin Institute of Technology’s Synonym Dictionary [46] is used to calculate the degree of similarity. The formula for cosine similarity is shown in Eq (1).


$$similarity\left(a,b\right)=cos\theta =\frac{\sum_{i=1}^{n}a_{i}*b_{i}}{\sqrt{\sum_{i=1}^{n}{\left(a_{i}\right)}^{2}}\times \sqrt{\sum_{i=1}^{n}{\left(b_{i}\right)}^{2}}}=\frac{a\cdot b}{\left|a\right|\times \left|b\right|}$$ (1)

Based on existing research, it is generally accepted that when the similarity score exceeds 0.8, the relationship names *a* and *b* are considered to belong to the same relationship and should be uniformly replaced.

#### Knowledge storage

Knowledge storage involves systematically organizing and storing the data that has undergone knowledge fusion, facilitating the development of the knowledge graph. The Qingyang sachet resources contain vast amounts of data, making the effective representation and storage of this data crucial for the application of the knowledge graph [28]. Typically, the visualization of a knowledge graph is presented in a graphical format, making the choice of storage method particularly important. There are three primary storage methods for knowledge graphs: RDF databases, traditional relational databases, and graph databases. Ontology querying based on graph databases is considered a more user-friendly and straightforward method [47]. Compared to traditional relational databases, Neo4j, a graph database specifically designed for graph data, is naturally compatible with the RDF data structure, allowing for the seamless representation and handling of RDF entities, properties, and relationships. Additionally, Neo4j offers efficient query performance, particularly for complex RDF data relationships. Therefore, this study utilizes the Neo4j graph database as the storage tool for Qingyang sachet knowledge, enabling the construction of the knowledge graph’s instances.

**Fig 6:**
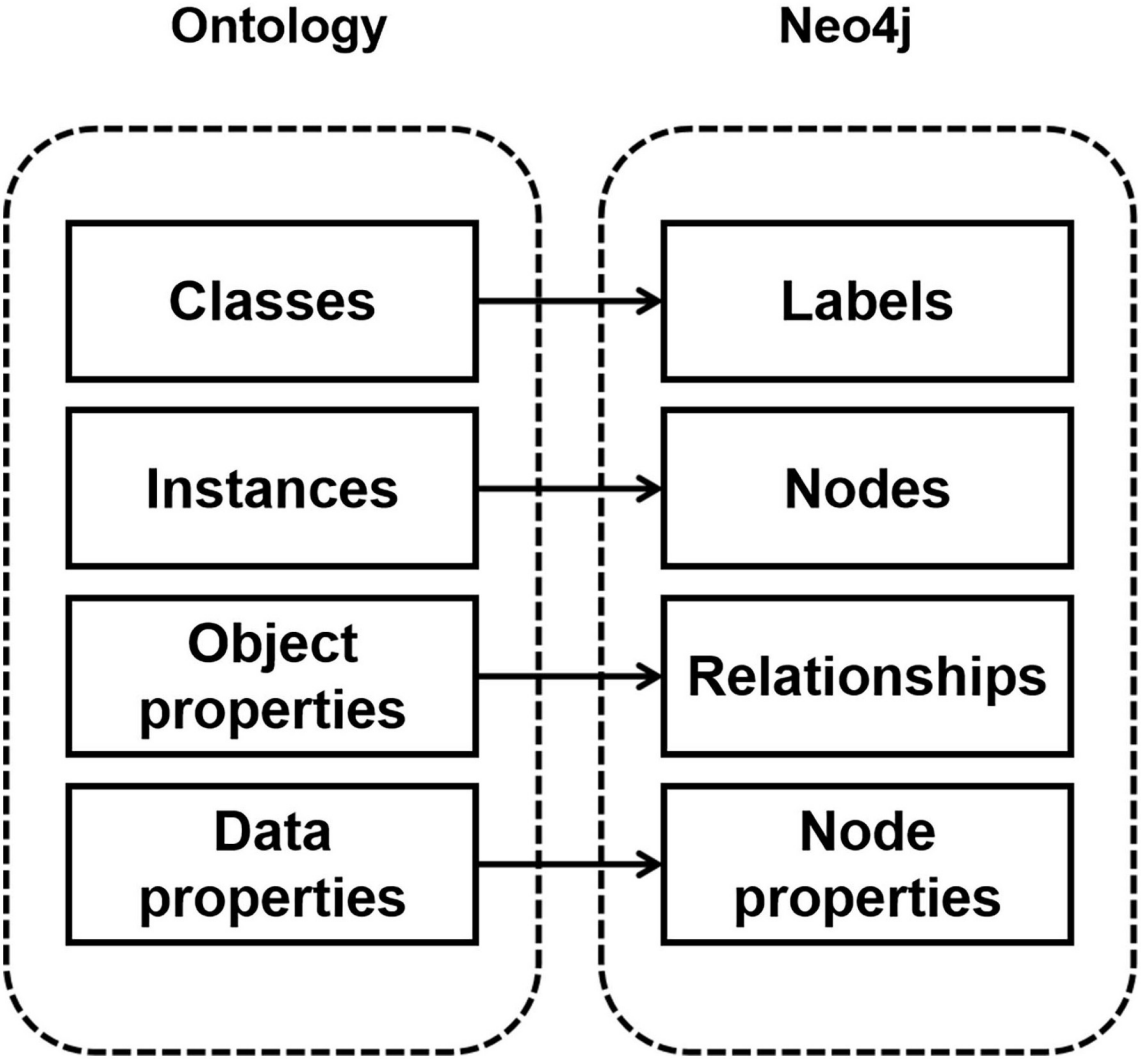
Ontology relationship mapping diagram.

In the Neo4j database, four elements—labels, nodes, relationships, and node properties—form the core structure, as shown in Fig 6. These elements correspond as follows: Classes are mapped to labels, instances to nodes, object properties to relationships, and data properties to node properties. This mapping completes the alignment from the Qingyang sachet ontology schema layer to the knowledge graph data layer. Neo4j primarily consists of nodes and edges. By examining the relationships between nodes and edges, the hierarchical structure of the ontology can be understood. The nodes represent the 10 core classes in the Qingyang sachet ontology model. The edges correspond to the 15 object properties in the ontology model, while the properties correspond to the 33 data properties.

During the storage process, the RDF framework established earlier is used to integrate “entity-relationship-entity” and “entity-property-property value” SPO triples, such as “Five Poison Toad—Craftsmanship—Person.” The data, in xlsx format, which contains all the established Qingyang sachet knowledge within the RDF framework, is batch imported into the Neo4j graph database. Adjustments are made to the properties of nodes and relationships, such as their color and size. As a result, this study constructs a multimodal knowledge graph of Qingyang sachets, which includes a total of 619 entity nodes and 1,003 relationships, covering various entities such as Qingyang sachets, time, places, people, events, craftsmanship, materials, patterns, functions, and cultural significance. The graph encompasses multidimensional information on Qingyang sachets, with high knowledge coverage and semantic richness. Using the Cypher query language, users can conduct semantic searches to retrieve relevant information, aiding them in better understanding and inheriting the sachet culture. This knowledge not only facilitates the digital preservation of sachet culture but also provides additional resources and inspiration for the design and application of sachets.

### Knowledge application based on the Qingyang sachet knowledge graph

#### Knowledge association

**Fig 7:**
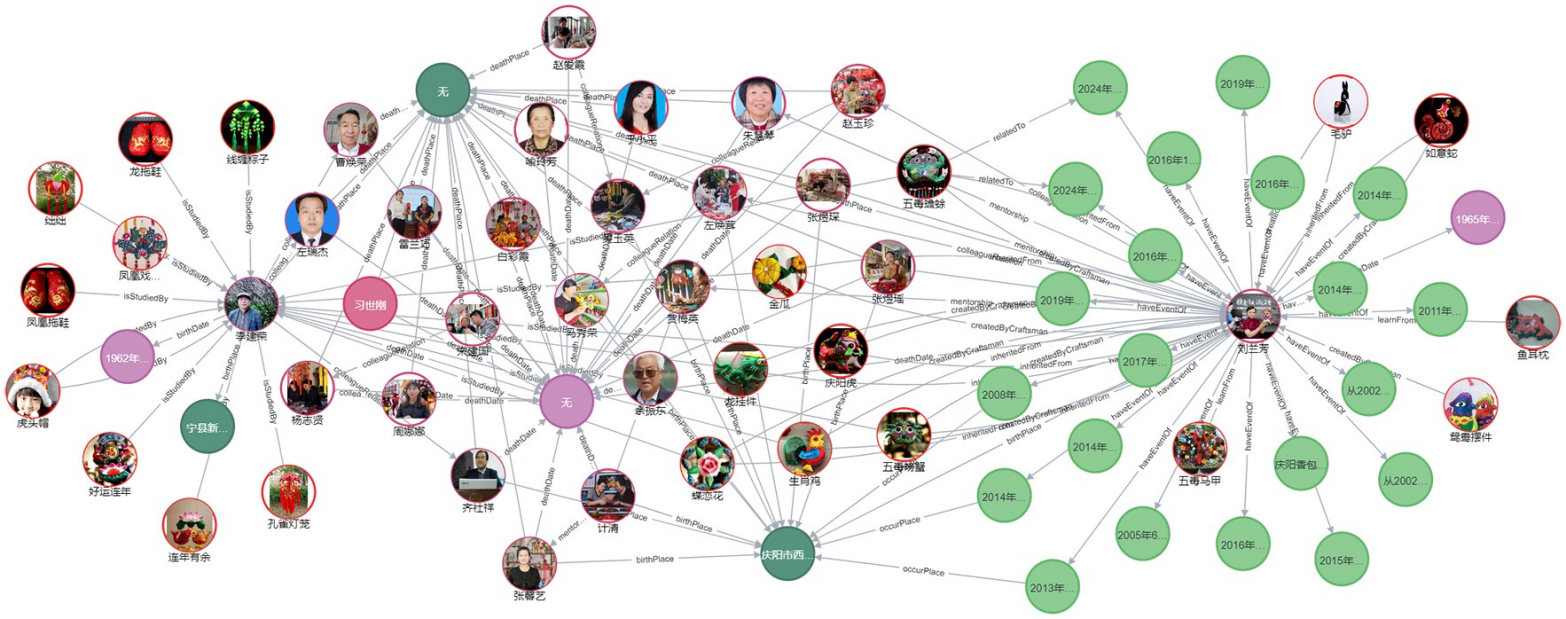
Visualization of the Qingyang sachet knowledge graph.

The visualization of the Qingyang sachet multimodal knowledge graph primarily involves presenting the relevant knowledge in a graphical format. The nodes and relationships within the graph are clearly defined, allowing users to adjust node sizes and view detailed node properties by clicking on the relevant modules. By analyzing the connections between edges, users can identify related properties among nodes, thereby gaining a more comprehensive understanding of the knowledge network associated with Qingyang sachets. Knowledge discovery, driven by various needs, involves extracting effective, comprehensive, and systematic knowledge from raw data. By entering relevant search terms during the knowledge graph operation, related knowledge units sharing the same properties can be systematically linked [48]. In this study, the instantiation of the Qingyang sachet knowledge graph presents data related to people, time, places, works, and other aspects associated with Qingyang sachets, thereby forming a knowledge network to uncover implicit knowledge and facilitate knowledge discovery. This approach transforms static and flat knowledge into a dynamic and multidimensional knowledge network. The overall visualization is shown in Fig 7 (some node information has been hidden for visual clarity). In this figure, red nodes represent the 33 types of Qingyang sachets studied in this research.

#### Knowledge retrieval

**Fig 8:**
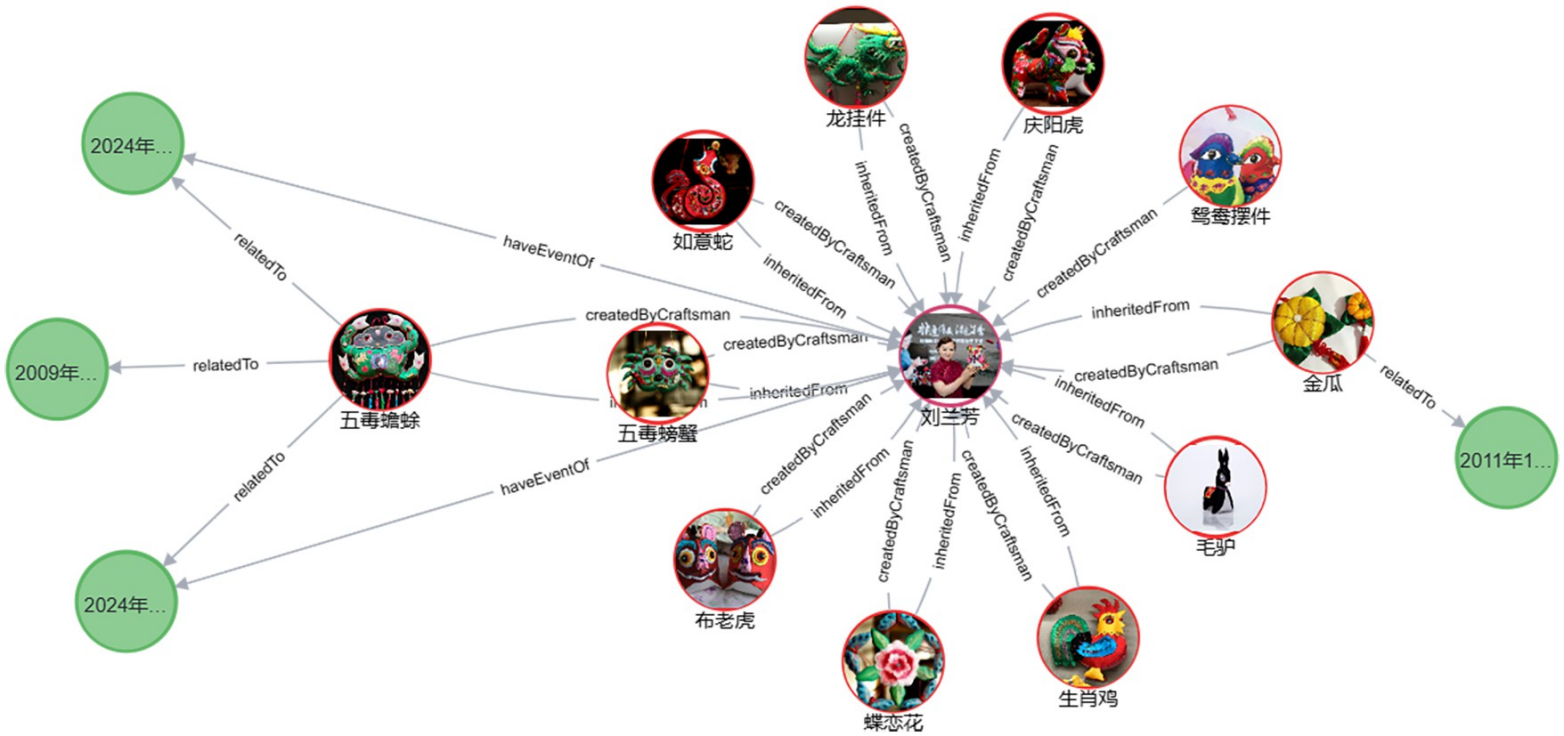
Knowledge retrieval based on ‘createdByCraftsman’.

Compared to structured query languages like SQL, Cypher, a query language optimized for graph databases, offers greater convenience. For example, when searching for “刘兰芳,” as shown in Fig 8, a Cypher query such as (match (b)-[:‘createdByCraftsmen’]->(a {name: “刘兰芳”}) return a, b) directly retrieves all related information. Lanfang Liu, born in Xifeng District, Qingyang City, is both a creator and inheritor of the Qingyang sachet. She has been primarily involved in the creation of 11 sachet works, including *Qingyang Tiger*, *Mandarin Duck Ornament*, *Golden Melon*, *Donkey*, *Butterfly Love Flower*, *Five Poison Crab*, and *Five Poison Toad*, all of which were crafted at the Qingyang Sachet Productive Protection Demonstration Base. In addition to her creative work, Liu has actively participated in the inheritance and promotion of Qingyang sachets. For instance, from July 6 to 10, 2024, she, along with her apprentice Yuyao Zhang, attended the 2nd China ICH Protection Conference in Yixing, Jiangsu Province, where the *Five Poison Toad* sachet was highly admired for its exquisite design and profound folk cultural significance. On July 20–21, 2024, Liu, with her apprentice Xinyue Liu, participated in the 30th Investment and Trade Fair in Lanzhou, Gansu Province. During the event, she demonstrated the process of making sachets, introduced the symbolism of the *Five Poison Toad* sachet as a talisman for warding off evil and seeking blessings, and shared stories of local artisans, conveying the unique charm of this traditional craft to attendees. Lanfang Liu also emphasized that sachet is not merely a piece of art but also a medium for passing down family bonds, affection, and cultural memories.

#### Knowledge discovery

**Fig 9:**
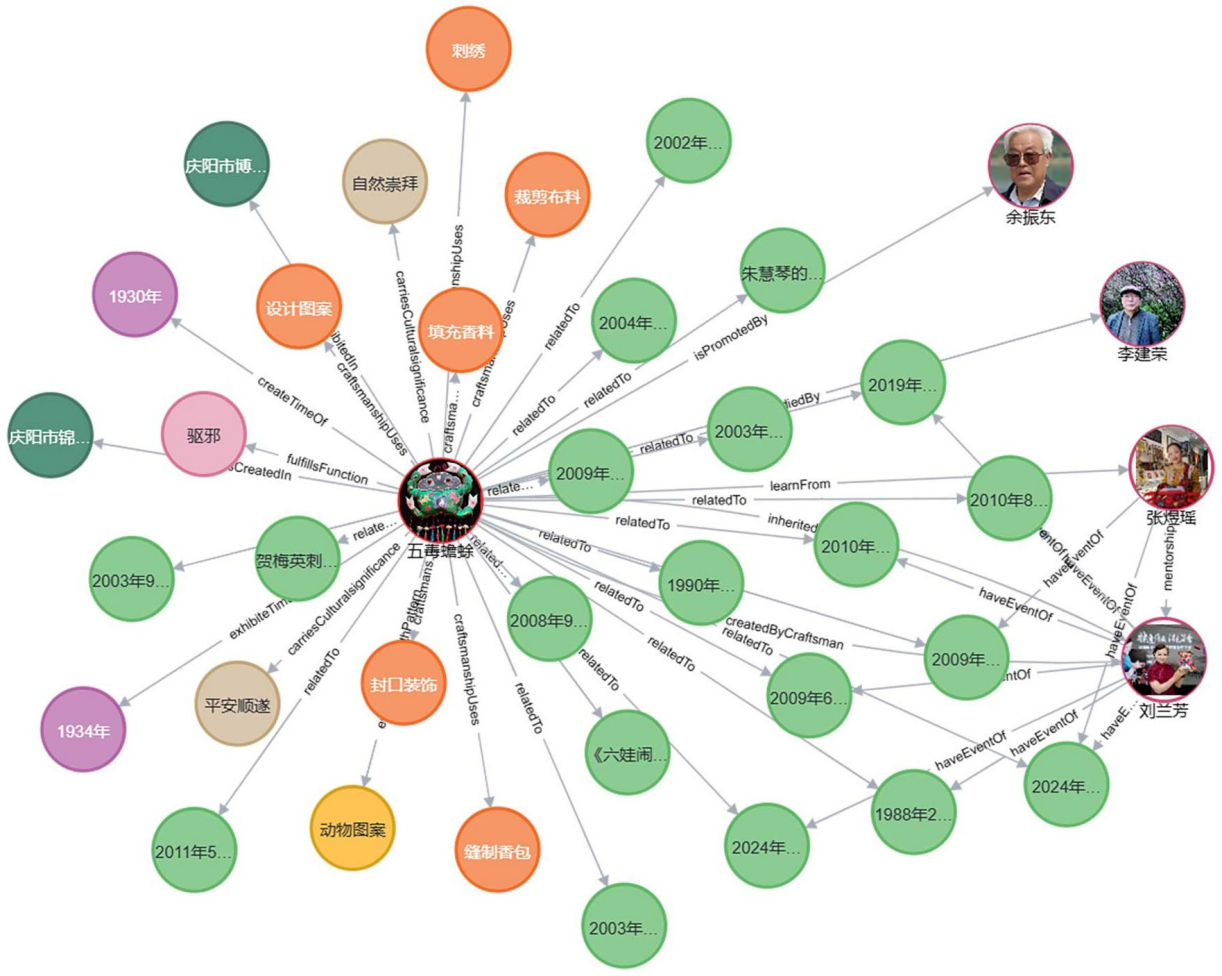
Knowledge discovery based on 五毒蟾蜍.

Knowledge discovery centered around Qingyang sachets allows for both a macro-level overview of the entire knowledge graph and a micro-level exploration of detailed information. Using the work *Five Poison Toad* as an example, this study demonstrates the process of querying, searching, and reasoning within the Qingyang sachet knowledge base, thereby facilitating knowledge discovery related to Qingyang sachets. As shown in Fig 9, by inputting the Cypher query (match (c:QingyangSachet {name: “五毒蟾蜍”})-[r*0‥]->(result) return result), a knowledge map centered on this work is generated, radiating outwards and providing a clear visualization of related information. The *Five Poison Toad* is a sachet pendant primarily in green, designed in the shape of a toad, with its back embroidered with five poisonous creatures: a snake, scorpion, centipede, gecko, and spider. This piece was created by Lanfang Liu in 1930 at the Jinxiu Workshop in Qingyang and is currently exhibited at the Qingyang Museum. Lanfang Liu’s apprentice, Yuyao Zhang, has inherited this craft, while Jianrong Li and Zhendong Yu have become researchers and promoters of *Five Poison Toad*. The creation of this sachet involves six main steps: designing the pattern, cutting the fabric, embroidering, filling with herbs, sealing, and decorating. By further exploring related nodes, users can access all information related to *Five Poison Toad*, uncovering hidden knowledge. For example, the sachet’s use extends beyond decoration; it is traditionally worn by children during the Dragon Boat Festival, symbolizing protection for their health and safety, warding off evil spirits and illness. In traditional Chinese beliefs, it is believed that during the Dragon Boat Festival, practices such as hanging mugwort and wearing sachets can ward off the harmful effects caused by the “Five Poisons.” Therefore, the *Five Poison Toad* sachet often features embroidered images of these animals, with the toad as a common motif. The *Five Poison Toad* vividly embodies the Chinese folk belief in “fighting poison with poison” and conveys a deep respect and reverence for nature. It also expresses a heartfelt wish for children’s healthy growth.

In summary, by constructing the Qingyang sachet knowledge graph, scattered information about ICH found in various literatures, oral histories, and practices can be systematically organized, forming a well-structured knowledge system. The digital knowledge graph ensures the long-term preservation of cultural heritage information, while also facilitating updates and maintenance. Even when certain skills or traditions are at risk of being lost, it provides a valuable reference for future restoration efforts. Moreover, the digital platform enables the public to easily access these precious cultural heritages. Regardless of geographical location, users can browse related information online, allowing them to quickly retrieve Qingyang sachet knowledge. The clear, intuitive visual presentation of this information helps users comprehend the entire body of knowledge related to a search term. Empowering knowledge discovery through the knowledge graph not only expands the scope of users’ knowledge searches but also uncovers users’ potential cognitive needs.

## Discussion and conclusion

Through the preliminary work of this study, it is evident that while digital research in the field of ICH has adopted knowledge graph technology, its application remains limited, particularly in studies focused on Qingyang sachet resources. This paper takes Qingyang sachets as the subject of study, designing an ontology model based on ten dimensions: works, people, time, place, events, craftsmanship, materials, patterns, functions, and cultural significance. Utilizing the Neo4j graph database, the paper constructs a multimodal knowledge graph of Qingyang sachets. Through knowledge retrieval and reorganization, latent connections were discovered, expanding the application of Qingyang sachet knowledge and enriching the research of digital humanities in the cultural field.

The construction of the Qingyang sachet knowledge graph presents multidimensional information, including craftsmanship, historical context, and cultural significance, in a visual format, providing a valuable resource for researchers, educators, and cultural enthusiasts. Compared to previously scattered, unstructured sachet data, the knowledge graph not only enhances the efficiency of data management and utilization but also provides systematic data support and a theoretical framework for the preservation and transmission of Qingyang sachets as ICH. Despite achieving some achievements, this study has several limitations. Firstly, the current knowledge graph is primarily based on literature and fieldwork. Future research could expand the data sources more deeply to enhance data diversity and comprehensiveness. Secondly, existing technologies and tools still face challenges in processing large-scale, multi-source data. Introducing more advanced natural language processing and machine learning techniques could improve data processing efficiency and accuracy. Lastly, in constructing the Qingyang sachet knowledge graph, we strictly adhered to ethical guidelines, ensuring that all aspects of the research met ethical requirements to safeguard its legitimacy and integrity. Future research and technological improvements could further refine the knowledge graph and expand its applications, such as establishing a dynamic update mechanism for the graph and designing multi-language, multi-platform user interfaces. More importantly, we intend to conduct real-world case studies to showcase the performance and value of the knowledge graph in practical applications. These efforts would contribute significantly to the preservation, transmission, and innovative development of ICH.
